# Pathophysiology of Type 1 Diabetes and Gut Microbiota Role

**DOI:** 10.3390/ijms232314650

**Published:** 2022-11-24

**Authors:** Federica Del Chierico, Novella Rapini, Annalisa Deodati, Maria Cristina Matteoli, Stefano Cianfarani, Lorenza Putignani

**Affiliations:** 1 Multimodal Laboratory Medicine Research Area, Unit of Human Microbiome, Bambino Gesù Children’s Hospital, IRCCS, 00165 Rome, Italy; 2 Diabetes & Growth Disorders Unit, Bambino Gesù Children’s Hospital, IRCCS, 00165 Rome, Italy; 3 Department of Systems Medicine, University of Rome Tor Vergata, 00133 Rome, Italy; 4 Department of Women’s and Children Health, Karolisnska Institute and University Hospital, 17177 Stockholm, Sweden; 5 Department of Diagnostic and Laboratory Medicine, Unit of Microbiology and Diagnostic Immunology, Unit of Microbiomics and Multimodal Laboratory Medicine Research Area, Unit of Human Microbiome, Bambino Gesù Children’s Hospital, IRCCS, 00165 Rome, Italy

**Keywords:** type 1 diabetes (T1D), insulin resistance, gut microbiome, dysbiosis, intestine permeability, butyrate production

## Abstract

Type 1 diabetes (T1D) is a multifactorial autoimmune disease driven by T-cells against the insulin-producing islet β-cells, resulting in a marked loss of β-cell mass and function. Although a genetic predisposal increases susceptibility, the role of epigenetic and environmental factors seems to be much more significant. A dysbiotic gut microbial profile has been associated with T1D patients. Moreover, new evidence propose that perturbation in gut microbiota may influence the T1D onset and progression. One of the prominent features in clinically silent phase before the onset of T1D is the presence of a microbiota characterized by low numbers of commensals butyrate producers, thus negatively influencing the gut permeability. The loss of gut permeability leads to the translocation of microbes and microbial metabolites and could lead to the activation of immune cells. Moreover, microbiota-based therapies to slow down disease progression or reverse T1D have shown promising results. Starting from this evidence, the correction of dysbiosis in early life of genetically susceptible individuals could help in promoting immune tolerance and thus in reducing the autoantibodies production. This review summarizes the associations between gut microbiota and T1D for future therapeutic perspectives and other exciting areas of research.

## 1. Introduction

Type 1 diabetes (T1D) is a multifactorial autoimmune disease characterized by T-cell-mediated destruction of insulin-producing β-cells of the pancreas. T1D is the most commonly diagnosed diabetes in children and adolescents (under 20 years of age) and causes ≥85% of all diabetes cases in these age groups worldwide [1]. Across the globe, it is evaluated that 1,110,100 people aged 0–19 years have T1D, with 128,900 newly diagnosed cases each year [2].

The condition is characterized by the body’s complete inability to produce insulin, an essential anabolic hormone that helps the body’s cells use glucose for energy. Importantly, insulin facilitates entry of glucose to muscle and adipose cells, induces the store of glucose as glycogen in liver and the synthesis of fatty acids, stimulates the uptake of amino acids, impedes the breakdown of fat in adipose tissue, and promotes the uptake of potassium into cells [3]. T1D is clinically characterized by the primary appearance of islet autoantibodies (AAbs). The AAbs are islet cell cytoplasmic antibodies (ICA), antibodies to insulin (IAA), to glutamic acid decarboxylase (GAD), insulinoma-associated 2 antibodies, or protein tyrosine phosphatase antibodies (IA-2) and zinc transporter8 antibodies (ZnT8) [4]. The natural history of T1D presents a “pre-stage 1” in which individuals carrying T1D susceptibility alleles have not yet developed islet AAbs. The development of two or more islet antibodies defines the “stage 1”, which can progress to “stage 2”, when dysglycemia appears, and then progresses to symptomatic diabetes (stage 3) [5]. The decline in β-cell number starts years before the symptoms of hyperglycaemia become evident. Moreover, a direct correlation exists between the number of detectable antibodies and their higher titers to the increased risk of developing T1D [3].

Recently, the development of T1D can be attributed to the intervention by genetic, epigenetic, and environmental factors. One such key identified environmental factor is gastrointestinal microbiota which comprises 100 trillion cells in the human gut, 10 times the number of human cells [6]. Since the gut microbiota plays a fundamental role in the revelation of framework and function of the host immune system, any changes in diet, overuse of antibiotics, or gastrointestinal tract infections can lead to substantial shifts in the composition of the individual microbiome over extensive periods. These shifts, which result in the disruption of the normal gut microbiota called the dysbiosis, lead to conditions such as autoimmune and inflammatory disorders [7,8]. Recent studies using high-end sequencing technologies have shown a significant difference in the intestinal microbial profile between T1D patients and healthy controls, suggesting correlation between T1D development and gut microbiota profile [9]. Moreover, new evidence proposes that perturbation in gut microbiota may be involved in the pathophysiology of T1D, and gut dysbiosis resulting in immunological deregulation and gut leakiness as the plausible pathogenic mechanisms in the onset of T1D [10]. Despite the latest findings, the explicit role of microbiota that mediates T1D is under investigation.

This review explores the link between microbiome, metabolism, and immune system, and focuses on their role in the development of T1D.

## 2. Epidemiology of T1D

Understanding the changing epidemiologic patterns of T1D, such as geographic differences, gender and age of the patients, and seasonal and ethnic factors in populations, can elucidate the incidence and prevalence of T1D.

T1D has previously been referred to as “juvenile diabetes” since it was found to be one of the most common chronic diseases of childhood, but lately T1D can be diagnosed at any age [11]. The condition occurs primarily between 5–7 years of age and at or near puberty [12]. T1D condition appears more commonly in males, exhibiting gender predominance, probably due to an estrogen protective role which is prominent with puberty [13]. The active role of estradiol in glucose homeostasis has been demonstrated by the increasing of insulin content and glucose-stimulated insulin secretion in isolated mouse pancreatic islets [14]. Moreover, its action on glycemia and insulin levels, glucose tolerance, and insulin secretion has been established in mouse models [15]. In an in vitro study, estradiol counteracted the effects of glucose in the induction of endoplasmic reticulum stress marker expression [16].

Interestingly, T1D occurrence was affected by season and birth months; T1D incidence is high in autumn and winter [17], whilst children born in the spring are associated with a higher chance of having T1D [18].

Across the world, the incidence and prevalence of T1D varies significantly. T1D is observed more commonly in European people, with Finland reporting >60 cases per 100,000 people each year following Sardinia, around 40 cases per 100,000 people each year [19]. Across the globe, the incidence of T1D is increasing by 3% every year, although profound reasons for this are unclear [20,21,22,23].

Globally, the presentation of T1D represents an epidemiological enigma, as wide variations in the disorder are reported between neighboring areas. For example, the incidence in Estonia suggests this conundrum, where T1D frequency is less than one-third of the incidence in Finland, although the two countries are separated by less than 120 km [24].

However, in the USA, from 2014–2015, annual T1D incidence rates for new cases are about 21 in 100,000. Furthermore, a more rapid increase in non-white racial and ethnic groups has been reported [25].

## 3. Pathophysiology of T1D

The pathophysiology of T1D seems to be far more complex than previously perceived. Research findings over the last two decades point at a complex interplay between several genetic, epigenetic, and environmental factors [26]. Though scientists agree that there is a strong immunological component in the progression of the disease, the exact triggering mechanisms still remain largely unclear. Studies on the natural history of T1D suggest that autoimmunity against multiple antigens of islet β-cells, driven by T-cells, is more likely to be triggered by environmental factors in genetically susceptible individuals [27]. Apart from the auto-immune component, recent findings also describe the role of β-cell-associated immunogenicity, which again could be attributed to certain genetic or environmental factors. In fact, the susceptibility of islet β-cells leads to cell stress and the formation of neoantigens that are attacked by the immune system [28].

Hence, a combination of various immune molecular events often results in local inflammation, known as insulitis, culminating in a marked and sustained loss of β-cell function and/or mass [27,29]. The timing of this destruction does not appear to be linear, but rather follows a relapsing-remitting kinetics similar to other autoimmune diseases [30]. In this context, the first signals of autoimmunity start long before the clinical evidence. In the very early stages, very few autoantigens are being recognized and the destruction of the islets is slowed down through mechanisms such as change in the surface antigens, allowing the pancreas to escape the autoimmunity attack [31,32]. The onset of hyperglycemia is caused both by the β-cells reduction and by their dysfunction. However, the range of this morphological and functional insufficiency varies amongst patients and concurs differently in the hyperglycemia development [33,34]. This indicates the possibility of small amounts of stable insulin production in some individuals with long-standing T1D, as demonstrated by longitudinal studies [34].

## 4. Role of Thymus-Dependent Immune System

The role of a Thymus-dependent immune system is well established in the development of the disease. During early ontogeny, inefficient thymic negative selection of self-specific T-cells is associated with several autoimmune diseases, including T1D [35,36]. Studies in non-obese diabetic (NOD) mice models revealed altered thymic development and Treg repertoire [37]. Tregs can develop in the thymus and pTregs develop mainly in the gut and can be primed by exposure to self-antigens and microbiome. In the thymus, certain self-reactive T-cells can escape negative selection and differentiate into Tregs [37]. Furthermore, experiments in mice have shown that Tregs specific to islet β-cell antigens are involved in T1D disease progression [38]. However, there remains a knowledge gap in the specific role of tTregs and pTregs in T1D development.

Contrary to the above theory of inefficient negative selection in T1D, significant studies report the presence of autoreactive T-cells against β-cells in blood, in similar frequencies, in both T1D patients as well as healthy individuals, suggesting an inherently flawed negative selection of β-cell antigens in all humans [28,39]. In accordance with these findings, Mallone et al. have proposed a state of “benign islet autoimmunity” [28]. Interestingly, the islet reactive T-cells in T1D patients were found to be of the memory T-cell phenotype [39,40,41]. Additionally, compared to healthy individuals, the frequency of T-cells in T1D patients was observed to be higher in the islets, suggesting a role of localized cues or β-cell immunogenicity in disease pathophysiology [28].

## 5. T1D Risk Factors

From a genetic standpoint, more than 50 gene loci have been implicated in T1D risk, most of which act on the immune system. By far, the strongest risk has been attributed to genes encoding human leukocyte antigen (HLA) genes [27]. Almost 90% of the patients were tested to carry the high-risk haplotypes DR4-DQ8 (DR4-DQA1*03:01-DQB1*03:02) or DR3-DRQ2 (DRB1*03:01-DQA1*05:01-DQB1*02:01) [42,43]. Several other immune-related genes have also been described in the pathogenesis of T1D [44,45]. Large-scale genome-wide association studies have identified multiple single nucleotide polymorphisms linked to T1D, many of them belonging to pathways involved in inflammation, immunity, and apoptosis [46]. Notably, there is often an overlap among T1D and other autoimmune and inflammatory traits loci. Some of these genes include *CTLA 4*, a down-regulator of the CD8+ T-cell response; *KIR* genes, a family of cell-surface receptors found on natural killer cells that regulate their function; interleukin (IL) genes such as *IL-4* and *IL-13*, which are immunomodulatory cytokines; *IL2RA*, interleukin-2 receptor subunit alpha, which is involved in Treg function [43,47].

Surprisingly, 90% of T1D cases have no first-degree relatives, and the pairwise concordance rate from homozygotic twins is described to be only 27% [48]. Furthermore, only 10–15% of the individuals with genetic risk ultimately develop T1D. This clearly indicates that the role of environmental triggers in the pathophysiology of T1D is much more significant than the one imputable to genetics. Moreover, the fact that T1D incidence has increased by several folds in the last 30 years, and the tendency of migrants to acquire the same risk of T1D as the population in their new area of residence, reinforces the hypothesis of the impact of environmental factors [49,50,51].

Numerous research findings describe a positive role of diet, lifestyle, gut microbiota, infections, and psychological stress in driving auto-immunity or β-cell dysfunction towards T1D development [51,52]. Among these, diet during early infancy could be an important factor. Breastfeeding is said to have a protective role, whereas the introduction of cow’s milk or cereals/gluten in early infancy could have an adverse role by inducing interferon (INF)-γ secretion and β-cell stress [52]. However, there are no conclusive human studies to prove this. Moreover, a protective role has been attributed to Vitamin D. As a modulator of inflammation, Vitamin D has a protective effect on IL-1-Th1-mediated damage of β-cells by inhibition of macrophage activation, abolition of CD4^+^ expression, inhibition of IL-2 and IFN γ, and reduction of the expression of major histocompatibility complex class II molecules [53]. Similarly, intake of omega-3 fatty is said to have a protective role in T1D inflammation [51].

In addition to these, certain viral infections, mainly enteroviruses (EVs), which enter through the intestine, have shown a strong positive correlation to T1D development [52]. One type of EV, the Coxsackievirus B, is known to replicate in islet β-cells and increase endoplasmic reticulum stress by disrupting the unfolded protein response pathway [28]. Finally, the gut microbiome composition has been observed to be different in healthy versus TID cases in both human and animal models.

## 6. Role of Gut Microbiota in T1D Pathophysiology

**Figure 1:**
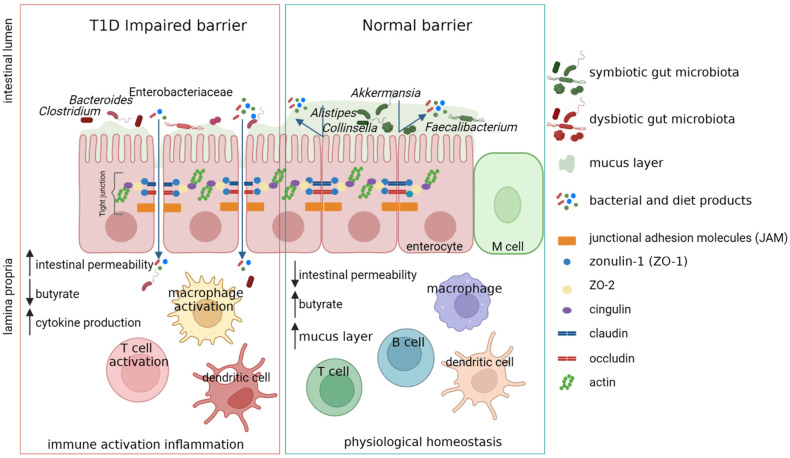
Increasing gut paracellular permeability in T1D patients. The alteration of tight junction (TJ) proteins leads to the increase of intestinal permeability, providing access to the lamina propria environment for foreign agents (e.g., bacteria and bacterial and diet products). The accumulation of these bacteria and molecules can trigger inflammation pathways, causing intestinal inflammation. The activated and expanded T-cells in the gut-associated lymphoid tissue (GALT) could travel via mesenteric and pancreatic lymph nodes to the pancreas and induce T1D. Created with BioRender.com.

A complex correlation between intestinal microbiota, immune system, and gut permeability has already been identified, although not completely unraveled [54]. The gut permeability is modulated by the gut barrier which comprises gut microbiota, mucus, enterocytes, tight junction (TJ) proteins, and the innate and adaptive immune cells forming the gut-associated lymphoid tissue [55]. The disassembly of TJ and the disruption of the integrity of the intestinal barrier can lead the intestinal permeability and the passage of microbial antigens and products or the microorganisms themselves. The TJ of the gut barrier is regulated by the expression of TJ proteins comprising claudin-2, occludin, cingulin, and zonula occludens (ZO) proteins. Some studies have demonstrated that the intestinal permeability depends on the increased levels of zonulin, whose production is influenced by bacterial colonization [56,57]. It is also understood that zonulin reversibly regulates intestinal permeability by modulating TJ [58,59,60]. Interestingly, before the onset of clinically evident T1D, high serum zonulin levels are present [61]. Moreover, in T1D patients, an increase of gut paracellular permeability has been detected [61,62,63,64,65] (Figure 1).

Interestingly, the increase of small intestinal permeability has also found in subjects at risk of developing T1D, confirming the hypothesis that alterations in intestinal mucosa barrier might be correlated to an autoimmune process that promotes disease onset [63,66]. Moreover, children with multiple islet autoantibodies (≥2 IA) who progressed to T1D presented a higher intestinal permeability compared those who did not progress, suggesting an involvement of intestinal permeability in the pathogenesis of T1D [67].

Many gut commensals play a role in modulating the permeability of the intestinal barrier [68]. A hypothesis is based on the evidence that some gut bacteria express GAD and produce gamma-aminobutyric acid. The GAD released from bacteria as a consequence of gut bacterial destruction (e.g., through viral- or antibiotic-mediated mechanisms) may act as an antigen to activate submucosal T-cells, causing miseducation of the host immune system and leading to the development of T1D [69,70].

Bioinformatics data confirmed that some of the microbes can carry peptide sequences similar to insulin, potentially triggering auto-immunity [71]. Interestingly, T-cell clones against preproinsulin peptides have shown high cross-reactivity to the peptides of *Bacteroides* and *Clostridium* species [72]. In a NOD mouse model, a peptide produced by *Parabacteroides distasonis* with homology to β-chain of insulin has been identified [73]. This peptide can be recognized by T-cells, cross-stimulating an immune response to this chain of insulin [73].

Lastly, by the gnotobiotic zebrafish model, it has been also demonstrated that the normal expansion of the pancreatic β-cell population during early larval development requires the intestinal microbiota by the activity of a bacterial protein termed β-cell expansion factor A (BefA), produced by gut microbes [74]. These findings shed light on a possible a role of the gut microbiota in early pancreatic β-cell development and suggest a link between fecal microbiota composition in childhood and an increase in diabetes risk.

## 7. Gut Microbiota Dysbiosis in T1D

The gut microbiota dysbiosis in T1D patients has already been described [75,76]. The “TEDDY study” showed the higher abundance of *Bifidobacterium* spp. and the lower abundance of *Streptococcus thermophilus* and *Lactococcus lactis* in children before the seroconversion or the onset of T1D with respect to healthy subjects [77].

The increase of *Bacteroides* abundance in T1D patients and subjects a risk to develop T1D compared to aged-matched healthy controls has been reported in different studies [75,78,79].

The alteration in microbiota composition seems to be present only in T1D progressors and not in children at risk who did not develop the disease. This evidence came from a Finnish study in which a reduction in microbiota richness was detected in children who develop T1D prior to diagnosis but after seroconversion [80]. Moreover, the seroconversion has been positively correlated with the increase of lipopolysaccharides biosynthesis in *Bacteroides* genome and sulfate reduction in *Anaerostipes* genome [81].

The enhanced activity of *Bacteroides* and a down-regulation of functions associated with *Bifidobacterium* in T1D patients were confirmed by a metaproteomic study on pediatric patients at T1D onset. In this study, the reduced activity of *Bifidobacterium* was highlighted in patients with low insulin need, suggesting the presence of a transient condition of the gut microbiota composition and functions related to a very early stage of the disease (Levi Mortera Stefano at al., “Functional and taxonomic traits of the gut microbiota in type 1 diabetes children at the onset: a metaproteomic study”. Submitted to International Journal of Molecular Sciences Manuscript ID: ijms-2032220, 30 October 2022).

In an Italian cohort of T1D patients, a high abundance *of Bifidobacterium stercoris*, *Bacteroides intestinalis*, *Bacteroides cellulosilyticus*, and *Bacteroides fragilis* was found [82]. An abundance of *Bacteroides dorei* and *Bacteroides vulgatus* was described in a cohort of Finnish children at high risk to develop T1D [83].

A study based on the integration of metagenomic and metabolomics approaches on T1D patients at onset, their siblings, and healthy subjects, revealed the increase in Clostridiales and *Dorea* and the decrease in *Dialister* and *Akkermansia* in T1D patients and their siblings, showing a specific profile of gut microbiota linked to familiar environment. Moreover, T1D patients were characterized by higher levels of isobutyrate, malonate, *Clostridium*, Enterobacteriaceae, Clostridiales, and Bacteroidales. Patients with higher anti-GAD levels showed low abundances of *Roseburia*, *Faecalibacterium*, and *Alistipes*, and those with normal blood pH and low serum HbA1c levels showed high levels of purine and pyrimidine intermediates. These results shed light on specific gut microbial and metabolic profiles predictive of T1D progression and severity [84].

Moreover, changes in the gut microbiota composition of T1D patients have been associated with glycemic control and disease-related complications, suggesting that the gut microbiota may also be involved in the development of diabetes-associated complications [85].

Moreover, in autoantibody-positive children, an increased abundance of *Bacteroides* and a low abundance of butyrate-producing species were found [76]. A negative correlation between butyrate-producers, the intestinal permeability, and the risk of developing T1D has also been reported [76,86,87,88]. However, even in the late phase of prediabetes, a low numbers of butyrate producers were found, suggesting the role of microbiota as a regulator of β-cell autoimmunity in the progression of the disease [10,76].

## 8. Butyrate-Associated Barrier Dysfunction in T1D Pathophysiology

Short chain fatty acids (SCFAs), such as acetate, propionate, and butyrate, are carbohydrate-derived metabolic products of certain bacterial commensals residing in the human intestine. SCFAs are known to exert wide-ranging beneficial effects that help maintain intestinal health and homeostasis. Therefore, dysbiosis and low SCFA production have implications in several diseases, including auto-immune diseases, cancer, pathogenic infections, cardiovascular disorders, and brain health [89,90,91]. Lately, butyrate has garnered immense attention and has been the main focus in microbiome-linked pathology studies. Although butyrate is less abundant, it has been identified as a crucial component involved in intestinal epithelial cell turnover, epithelial barrier function, energy production, and immunological pathways [90,92].

Butyrate production is mediated by gut microbiota through the anerobic fermentation of dietary starch and fiber, and sometimes proteins [90,92]. Identifying butyrogenic configurations of microbiota is challenging, as several bacterial species are involved in a multistep mechanism in the production of butyrate.

The most abundant producers present in the colon belong to the *Bacteroides*, *Clostridium* cluster XIVa, *Clostridium* cluster IV groups [93], *Eubacterium Rectale* from Ruminococcaceae family (Clostridial cluster XIVa), *Faecalibacterium prausnitzii* from Lachnospiraceae family (Clostridial cluster IV), Erysipelotrichaceae, and Clostridiaceae [94].

Numerous pathways have been described for the synthesis of butyrate by gut microbes. To begin with, the polysaccharides in the food are first converted to acetoacetyl-CoA via glycolysis, which is then reduced to butyryl-CoA and finally converted in butyrate [92,94]. Moreover, several species of *Firmicutes*, *Fusobacteria*, and *Bacteroidetes* are capable of producing butyrate from peptides and amino acids, principally via the lysine and glutamate pathways [94]. Recently, in addition to these direct pathways, several research findings highlight the concept of cross-feeding among various bacterial species, wherein primary degraders ferment polysaccharides into intermediary metabolites such as lactate and acetate, which are further metabolized by secondary degraders into different molecules such as butyrate [94]. Thus, butyrate production in a normal healthy gut can be summarized as a result of a complex metabolic network involving multiple bacterial communities, and likely other microbial species, which remains to be elucidated.

Metagenomic studies reveal a significant reduction in the number of butyrate-producing species from *Clostridium* clusters IV and XIVa, as well as mucin-degrading bacteria such as *Prevotella* and *Akkermansia* in T1D patients [84,87].

Thus, butyrate, through its activity on gut permeability, can play an important role in the progression of T1D. However, the consequences of gut permeability and the cascade of molecular events that lead to progression of T1D is just beginning to unfold. Interestingly, the administration of butyrate has shown up to 30% remission of T1D in NOD mice [95]. In addition, treatment with sodium butyrate has also shown to improve insulin resistance and related metabolic disorders [96].

In summary, although burgeoning evidence point at a prominent connection between the loss of butyrate-producing species, gut permeability, and T1D disease progression, further focused studies should be designed to elucidate the precise molecular underpinnings in these contexts.

## 9. Probiotics-Based Treatment in T1D Patients

Starting from the evidence that correcting dysbiosis in early life could help to promote immune tolerance and thus inhibit the initiation of β-cell autoimmunity, Ziegler and colleagues proposed a daily administration of *Bifidobacterium infantis* in newborns from 7 days old to 12 months old with an elevated genetic risk for T1D to determine whether the cumulative incidence of β-cell autoantibodies in childhood could be reduced [97]. To date, the patients’ recruitment of this trial is still open (https://clinicaltrials.gov/ct2/show/NCT04769037 (accessed on 29 September 2022)).

In a double-blind and randomized placebo-controlled trial, children with newly diagnosed T1D were supplemented with probiotics for three months. In the treated patients, HbA1c and insulin bolus doses decreased significantly compared with those of the placebo group, with no adverse reactions reported [98].

Finally, in a randomized controlled trial, allogenic vs. autologous fecal microbiota transplantation (FMT) was used in recent onset T1D patients. After 12 months from FTM, a preserved stimulated c-peptide levels and several novel bacterial strains in the autologous FTM group were found. Moreover, a change in small intestinal CCL22 expression and whole blood immune cell subsets, such as CXCR3+ CD4+ T-cells, have been reported, confirming a potential role of microbiota in T1D development and progression [99].

However, not all the studies based on the probiotic supplementation on T1D have been successful. For instance, the supplementation of *Lactobacillus rhamnosus* GG and *Bifidobacterium lactis* Bb12 had no significant effect in maintaining the residual pancreatic β-cell function in children with newly diagnosed T1D [100].

It remains to be defined which microbial-based treatments are potentially the most favorable for management of T1D patients, but also in which phase of T1D they are most effective.

## 10. Conclusions

Although T1D was earlier regarded to have genetic roots, compelling evidence states a strong role of environmental factors in disease onset and progression. In this context, several studies report gut dysbiosis in T1D patients with a marked decrease in butyrate-producing communities. Among the various physiological roles of butyrate in the gut, its role in maintaining the integrity of the gut mucosal barrier has been highlighted. A permeable gut barrier allows contact between immune cells and the residing microbes, disrupting the fine line between commensalism and pathogenicity and confirming the protective role of butyrate in preventing T1D.

Therefore, from a therapeutic point of view, microbiota-based therapy to slow down disease progression or reverse T1D has been gaining prominence. The inclusion of probiotics and prebiotics (carbohydrates and dietary fiber) in the diet can confer a protective effect by improving gut permeability and decreasing inflammation. Finally, FMT seems to be a promising treatment to decline the endogenous insulin production and to preserve residual β-cell function.

Collectively, all these points emphasize how the gut microbiome in the early years of life can influence host metabolism and lay foundations for health complications later in life.

## Data Availability

Not applicable.
